# Analysis of Factors Associated with the Severity of Acute Pancreatitis according to Etiology

**DOI:** 10.1155/2017/1219464

**Published:** 2017-12-07

**Authors:** Dae Bum Kim, Woo Chul Chung, Ji Min Lee, Kang-Moon Lee, Jung Hwan Oh, Eun Jung Jeon

**Affiliations:** ^1^Division of Gastroenterology, Department of Internal Medicine, St. Vincent Hospital, The Catholic University of Korea, Suwon, Republic of Korea; ^2^Division of Gastroenterology, Department of Internal Medicine, St. Paul's Hospital, The Catholic University of Korea, Seoul, Republic of Korea

## Abstract

**Background:**

The objective of this study was to determine the factors associated with severity of acute pancreatitis (AP) according to two major etiologies: alcohol and gallstones.

**Methods:**

We reviewed the medical records of consecutive patients who were admitted with AP between January 2003 and January 2013. A total of 905 patients with AP (660 alcohol-induced, 245 gallstone-induced) were enrolled. Among them, severe AP (SAP) occurred in 72 patients (53 alcohol-induced, 19 gallstone-induced). Contributing factors between patients with and without SAP were analyzed according to the etiology.

**Results:**

Multivariate analysis demonstrated that current smoking, pancreatic necrosis, and bacteremia were associated with AP severity in both alcohol- and gallstone-induced AP. Pancreatic fluid collection was significantly associated with alcohol-induced SAP (*p* = 0.04), whereas dyslipidemia was significantly associated with gallstone-induced SAP (*p* = 0.01). Body mass index was significantly correlated with the Bedside Index of Severity in Acute Pancreatitis score in both alcohol- and gallstone-induced AP (*p* = 0.03 and 0.01, resp.).

**Conclusions:**

Current smoking, pancreatic necrosis, and bacteremia can aggravate the clinical course of AP. Pancreatic fluid collection and dyslipidemia were associated with AP severity according to the different etiologies. Obesity may also be associated with AP severity in both etiologies.

## 1. Introduction

Acute pancreatitis (AP) is a sudden inflammatory condition of the pancreas. Although conservative management results in clinical improvement in most patients, approximately 5% to 10% of cases progress to life-threatening conditions, including multiorgan failure, with significant morbidity and mortality [1]. There is an increasing trend in the annual incidence of AP in Western countries and Korea [2–4]. Of the various etiologies of AP, the two most common are gallstones and alcohol abuse, which represent more than 60% of all cases [2, 5]. Gallstone- and alcohol-induced AP should be treated as distinct entities, and the different etiologic factors may affect the clinical outcome, severity, and recurrence. A better understanding of the etiology is directly linked to more favorable outcomes and is important for the establishment of treatment strategies and prevention of recurrent AP.

Severe AP (SAP) is associated with relatively high morbidity and mortality rates [6–8]. The Acute Physiology and Chronic Health Evaluation scale (APACHE II), Bedside Index of Severity in Acute Pancreatitis (BISAP), Ranson's criteria, Glasgow criteria, and computed tomography severity index are risk stratification systems for the prediction of the clinical course and prognosis of AP. Generally, APACHE II is the most widely used method for risk stratification in AP, while BISAP is an uncomplicated system with a score that is easy to calculate [9, 10]. Except for modified Ranson's criteria, the other scoring systems have the same severity scoring parameters generally, regardless of the etiology of AP. In the modified Ranson scoring system, the variables to define severity are dissimilar according to etiology [11]. For nongallstone-induced AP, the parameters are more stringent compared with gallstone-induced AP. In practice, the majority of patients with gallstone-induced AP have a mild clinical course. In this point of view, different risk factors might contribute to AP severity based on different etiologies. For example, obesity is considered to be an independent risk factor for SAP [12, 13], though there has been a conflicting report [14]. Especially in Asian populations, worse clinical outcomes tend to occur in patients with low body mass index (BMI) [15, 16].

In the present study, we determined the factors associated with severity of AP according to etiology and evaluated the relationship between obesity and AP severity in the Korean population.

## 2. Methods

### 2.1. Study Population

This study was conducted at St. Vincent Hospital (Suwon, Korea) and St. Paul's Hospital (Seoul, Korea), The Catholic University of Korea. We retrospectively reviewed the medical records of consecutive patients who were admitted with AP between January 2003 and January 2013. Every patient underwent abdominal computed tomography, ultrasonography, or magnetic resonance imaging of the pancreas. After etiologic evaluation, we included patients who had gallstone- or alcohol-induced AP.

### 2.2. Definition

Diagnosis of AP was established by the presence of 2 of the following 3 criteria: (1) abdominal pain compatible with the disease, (2) increased serum amylase and/or lipase (>3 times the upper limit of normal), and/or (3) characteristic features on abdominal imaging studies.

Gallstone-induced AP was diagnosed if the patient had a gallstone, sludge in the gallbladder, and/or lithiasis in the common bile duct with or without a dilated bile duct observed in imaging studies. Alcohol-induced AP was defined as alcohol consumption just before the development of AP without any other cause of pancreatitis. Patients with acute exacerbation of chronic pancreatitis were excluded.

SAP was defined by the Atlanta consensus classification system based on persistent organ failure, including cardiovascular failure, respiratory insufficiency, or renal insufficiency [17]. Various risk factors possibly related to SAP, including sex, smoking, alcohol consumption, necrosis, pancreatic fluid collection, bacteremia, dyslipidemia, diabetes mellitus, and BMI on admission, were analyzed according to each etiology.

As for the evaluation of AP severity related to obesity, all enrolled patients were assessed according to BISAP score.

### 2.3. Statistical Analysis

Continuous data were expressed as mean ± SD and analyzed using the independent samples *t*-test or Kruskal-Wallis test. Categorical variables were expressed as quantities and analyzed using the *χ*^2^ test or Fisher's exact test. Logistic regression analysis was performed to identify the independent factors associated with SAP. Simple regression analysis was performed to evaluate the correlation between BMI and AP severity based on BISAP score. SPSS version 18.0 (SPSS Inc, Chicago, IL, USA) was used for the analyses, and a *p* value of <0.05 was considered statistically significant.

### 2.4. Ethical Consideration

This study was reviewed and approved by the Institutional Review Board of The Catholic University of Korea (VC15RISE0185) and was in compliance with the Declaration of Helsinki.

## 3. Results

### 3.1. Etiologic Analysis

A total of 905 patients with AP were enrolled. The frequency of alcohol-induced AP was 72.9% (660/905), and a male-predominant pattern (528 males, 132 females) was seen in this type of AP. The mean age of patients with alcohol-induced AP was 51.40 ± 14.60 years. The frequency of gallstone-induced AP was 27.1% (245/905), and a male-predominant pattern (154 males, 91 females) was also seen in this type of AP. The mean age of patients with gallstone-induced AP was 59.51 ± 15.57 years. SAP occurred in 72 patients (53 alcohol-induced, 19 gallstone-induced). Pancreatitis-related death occurred in 19 patients (18 alcohol-induced, 1 gallstone-induced) (Table 1).

### 3.2. Multivariate Analysis According to Etiology

In multivariate analysis, current smoking, pancreatic necrosis, and bacteremia were significantly (*p* < 0.05) associated with SAP in both alcohol- and gallstone-induced AP (Tables 2 and 3). Pancreatic fluid collection was significantly associated with alcohol-induced SAP (*p* = 0.04) but not gallstone-induced SAP (*p* = 0.27). Dyslipidemia was significantly associated with gallstone-induced SAP (*p* = 0.01) but not alcohol-induced SAP (*p* = 0.07).

**Figure 1:**
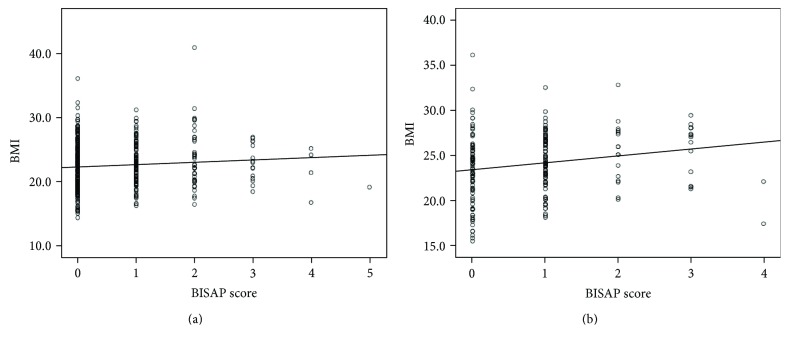
Simple regression analysis of body mass index (BMI) and Bedside Index of Severity in Acute Pancreatitis (BISAP) score. In both (a) alcohol-induced pancreatitis (*p* = 0.03) and (b) gallstone-induced pancreatitis (*p* = 0.01), there was a significant correlation between BMI and BISAP score.

When patients were classified according to BMI as obese (BMI ≥ 25 kg/m^2^) or nonobese (BMI < 25 kg/m^2^), obesity was not significantly correlated with severity in alcohol- or gallstone-induced AP. However, simple linear regression analysis revealed that BMI was correlated with the BISAP score in both alcohol- and gallstone-induced AP (*p* = 0.03 and 0.01, resp.; Figure 1).

## 4. Discussion

We performed a retrospective analysis of patients with AP during a recent decade and investigated the factors associated with severity of AP according to etiology. In this study, SAP was defined by the Atlanta consensus classification, which is based on organ failure [17]. However, we found that current smoking, pancreatic necrosis, and bacteremia could aggravate the clinical course of AP.

Smoking emerged as an independent factor associated with AP. Several experimental animal studies have suggested that exposure to smoking can induce pathologic and functional changes in the pancreas that can cause inflammatory activity [18–21]. Smoking can also elevate the level of pancreatic zymogens in the blood stream after secretin stimulation [22]. In multivariate analysis, we found that smoking was an independent factor associated with SAP in both alcohol- and gallstone-induced AP.

In addition, our results showed that pancreatic necrosis and bacteremia aggravated the clinical course of both alcohol- and gallstone-induced AP. A significant correlation has been demonstrated between the extent of pancreatic necrosis and organ failure. Previous reports have suggested that 5% to 10% of patients with AP develop pancreatic necrosis [17, 23]. The extent of pancreatic necrosis is associated with infection of pancreatic tissue, which augments the inflammatory cascade, resulting in organ failure [24–26].

Moreover, AP is frequently complicated by infection, such as pneumonia, bacteremia, and infected pancreatic necrosis. Among them, bacteremia has been reported to be a risk factor for infected pancreatic necrosis and a high mortality rate [27]. Consistent with previous studies, bacteremia was associated with SAP in this study. Pancreatitis may contribute an inflammatory change in the gastrointestinal tract, causing bacterial translocation from the intestinal lumen to the blood stream. Bacteremia is a common pathomechanism to explain multiorgan failure in severely ill patients and, thus, could be a factor associated with SAP.

Our study also demonstrated that pancreatic fluid collection was associated with SAP in alcohol-induced AP. Previous studies have shown that the incidence of pseudocyst formation or peripancreatic fluid collection was higher in patients with alcohol-induced AP compared with gallstone-induced AP [28, 29]. Alcohol-induced AP usually develops in heavy alcoholics who have already developed underlying pancreatic injury at the time of AP diagnosis [30]. Therefore, they are more vulnerable to pancreatic fluid collection following pancreatic ductal damage. For these reasons, pancreatic fluid collection is not only common in alcohol-induced AP but might also relate to the severity of AP.

This study found that dyslipidemia was a risk factor for SAP in gallstone-induced AP but not alcohol-induced AP. To date, the correlation between dyslipidemia and severity of AP remains ambiguous. Although no significant correlation has been observed between triglyceride levels and AP [31], some reports have described that higher triglyceride levels were associated with a more severe prognosis of acute biliary pancreatitis [32, 33]. It has been hypothesized that elevated triglyceride levels could lead to increased blood viscosity, which can impair pancreatic blood circulation [32]. A large amount of free fatty acids generated in pancreatitis can promote the burden on the endoplasmic reticulum through cholecystokinin expression [34, 35]. Furthermore, low high-density lipoprotein cholesterol levels, which have known anti-inflammatory properties, can cause a more severe systemic response [36, 37].

Although the precise mechanisms have not been elucidated, obesity is a possible independent predictor of AP outcome [38, 39]. In several previous studies, obese patients demonstrated decreased pancreatic microcirculation compared with nonobese patients and presented with more severe pancreatic inflammation and necrosis, subsequently leading to local infection [12, 40, 41]. Multivariate analysis in our study showed that obesity was not significantly associated with SAP. However, obesity did exhibit a linear correlation with BISAP score. Further large-scale studies would be required to draw concrete conclusions.

This study has several potential limitations. First, its design was retrospective. However, we tried to overcome this drawback by obtaining consecutive data using a standard approach and enrolling a large number of patients. Second, BMI was used as an indicator of the quantity of body fat. In addition to BMI, visceral adipose tissue measured using the waist-to-hip ratio and waist circumference has been proposed as a risk factor associated with worse outcomes. Fat depots close to the pancreas are known to be commonly affected by pancreatitis. In clinical practice, peripancreatic fat necrosis as a part of necrotizing pancreatitis correlates with worse outcomes of AP [42]. However, we did not evaluate the waist-to-hip ratio or waist circumference.

## 5. Conclusion

The results of our study revealed that current smoking, pancreatic necrosis, and bacteremia could aggravate the clinical course of AP, while both pancreatic fluid collection and dyslipidemia were associated with AP severity according to different etiologies. Further nationwide studies are needed to validate these findings.

## Figures and Tables

**Table 1:** Baseline characteristics.

	Alcohol	Gallstones	*p* value
No. of patients (%)	660 (72.9)	245 (27.1)	
Sex, M : F, *n*	528 : 132	154 : 91	<0.01
Age, mean ± SD, y	51.40 ± 14.60	59.51 ± 15.57	<0.01
BMI, mean ± SD, kg/m^2^	22.01 ± 5.29	24.04 ± 3.51	0.01
AP reattack, *n*	171	54	0.23
SAP, *n*	53	19	0.89
Pancreatitis-related death, *n*	18	1	0.03

AP: acute pancreatitis; BMI: body mass index; SAP: severe acute pancreatitis.

**Table 2:** Multivariate analysis of factors associated with severe acute pancreatitis induced by alcohol consumption.

	OR	95% CI	*p* value
Smoking	2.59	0.99–7.19	0.05
Pancreatic necrosis	10.46	4.20–26.05	<0.01
Bacteremia	11.68	3.13–43.60	<0.01
Pancreatic fluid collection	2.85	1.00–8.10	0.04

CI: confidence interval; OR: odds ratio.

**Table 3:** Multivariate analysis of factors associated with severe acute pancreatitis induced by gallstones.

	OR	95% CI	*p* value
Smoking	7.22	1.05–49.69	0.04
Pancreatic necrosis	8.95	0.97–82.25	0.05
Bacteremia	10.71	2.04–56.12	<0.01
Dyslipidemia	5.21	1.36–19.94	0.01

CI: confidence interval; OR: odds ratio.
